# ‘One woman, one bed’: prevalence and factors associated with women’s experiences of respectful birth in urban Dar es Salaam, Tanzania – across-sectional survey

**DOI:** 10.1080/16549716.2025.2568295

**Published:** 2025-10-24

**Authors:** Brenda Sequeira D’mello, Natasha Housseine, David Sando, Johnson Mshiu, Zainab Muniro, Evance Polin, Nuswe Ambokile, Hudson August, Nanna Maaløe, Jos van Roosmalen, Thomas van den Akker, Maembe Luzango, Idrissa Kabanda, Mtingele Sangalala, Tarek Meguid, Dan Wolf Meyrowitsch, Hussein Lesio Kidanto

**Affiliations:** a Maternal and Newborn Healthcare, Comprehensive Community Based Rehabilitation in Tanzania (CCBRT) Institution, Dar es Salaam, Tanzania; b Global Health Section, Department of Public Health, University of Copenhagen, Copenhagen, Denmark; c Medical College, East Africa, Aga Khan University, Dar es Salaam, Tanzania; d Management and Development for Health (MDH), Dar Es Salaam, Tanzania; e National Institute for Medical Research, Dar es Salaam, Tanzania; f Temeke Regional Referral Hospital, Department of Obstetrics and Gynaecology, Dar es Salaam, Tanzania; g Department of Obstetrics and Gynecology, Copenhagen University Hospital, Hvidovre, Denmark; h Athena Institute, VU University, Amsterdam, The Netherlands; i Department of Obstetrics and Gynaecology, Leiden University Medical Center, Leiden, The Netherlands; j Department of Obstetrics and Gynaecology, Mwananyamala Regional Referral Hospital, Dar es Salaam, Tanzania; k Sinza (Maternity) Hospital, Ubungo Municipal, Presidents Office, Regional and Local Government, Dar es Salaam, Tanzania; l Department of Obstetrics and Gynaecology, Amana Regional Referral Hospital, Dar es Salaam, Tanzania; m Department of Paediatrics and Child Health, University of Cape Town, Cape Town, South Africa

**Keywords:** person-centered-maternity care, Respectful Maternity Care, rights-based care, disrespect and abuse, obstetric violence

## Abstract

**Background:**

Respectful maternity care (RMC) is essential for quality care, safety, and a fundamental right of women during childbirth. However, mistreatment during childbirth hinders global efforts to reduce maternal and perinatal deaths and birth-related injuries. In rapidly urbanizing Dar es Salaam, disrespectful care in overcrowded maternity units is concerning.

**Objective:**

To assess the prevalence and factors associated with women’s experiences of RMC in four urban health facilities in Dar es Salaam.

**Method:**

A 25-item locally co-created and validated measurement tool was administered to 838 postnatal women before discharge in a cross-sectional survey. Data were analyzed in Stata 14 to describe sociodemographic characteristics, birth outcomes, and birth experiences. Multivariable logistic regression identified factors associated with RMC.

**Results:**

Satisfaction was reported by 96.4% (793/823) of women. Additionally, 84.3% (689/817) reported effective communication. However, 60.8% (503/827) shared hospital beds, 32.2% (253/785) experienced mistreatment, and 10.7% (89/829) had a birth companion. RMC was significantly less frequent among single women (aOR 0.56; 95% CI: 0.36–0.87) and those with childbirth complications (aOR 0.52; 95% CI: 0.35–0.78). Complications were reported less frequently when women had their own bed (aOR 0.51; 95% CI: 0.34–0.77).

**Conclusion:**

High satisfaction scores, despite mistreatment, bed-sharing, and lack of birth companionship highlight the need to raise awareness of rights-based care in communities. As urban growth strains healthcare systems, addressing structural constraints and overcrowding is crucial. Strengthening provider training in RMC and complications management, along with institutionalizing RMC measurements, can improve accountability, clinical outcomes, and women’s experiences of care.

## Paper Context

● **Main findings:** A survey of 838 postnatal women in four urban maternity units in Dar es Salaam using a validated Respectful Maternity Care tool found high satisfaction (96.4%) and effective communication (84.3%); however, two-thirds shared hospital beds, one-third experienced mistreatment, and those with birth complications had higher odds of mistreatment and sharing beds.● **Added knowledge:** The gap between high satisfaction and widespread mistreatment, including bed-sharing indicates that satisfaction surveys overestimate care quality and highlight the importance of measuring satisfaction and specific mistreatment experiences.● **Global health impact for policy and action:** Advocacy for institutionalizing measurement of respectful care, and further exploration of the link between overcrowding, birth complications, and mistreatment is essential.

## Background

Respectful Maternity Care (RMC) is an integral component of quality maternal health care, rooted in professional codes of conduct, bioethics and universal rights [1–3]. According to the World Health Organization (WHO), RMC not only upholds women’s dignity, privacy and confidentiality, but also ensures protection from harm and mistreatment, promotes informed choice and provides continuous labor support [4]. Positive facility-based birth experiences are essential to maintain high institutional birth rates and reduce maternal and perinatal mortality and morbidities as countries strive to achieve the Sustainable Development Goals established by the United Nations in 2015 [1,5]. Despite increased institutional births, global maternal and perinatal mortality rates remain stagnant, highlighting concerns about the quality of care and mistreatment during childbirth [6–9].

Acknowledging the epistemic value of ‘obstetric violence’ in highlighting systemic reproductive oppression, this author group uses ‘mistreatment’ in line with WHO terminology [4,10,11]. This choice does not diminish the severity of these violations or women’s right to RMC. As outlined in our previous paper, ‘RMC’, as commonly understood in Tanzania, refers to rights-based care in a supportive, nurturing environment and the absence of mistreatment [1,12].

Mistreatment during childbirth is a widespread issue, affecting both high-income and low-income countries, with global prevalence rates often exceeding 40% [13–19]. Factors such as patient characteristics (adolescent age, single, HIV-positive, ethnic background), provider attributes (lack of skills and professional ethics), facility conditions (ill-equipped or overcrowded), and systemic issues (lack of resources and poor leadership) contribute to mistreatment during childbirth [20,21]. In low- and middle-income countries (LMICs), mistreatment often includes more overt actions such as humiliation, physical abuse, neglect and verbal insults, whereas high-income countries typically report subtler forms of mistreatment, often affecting marginalized groups or manifesting as limited autonomy in care decisions [22].

In Tanzania, reported mistreatment during facility-based childbirth varies by interview type, scale items, computation methods, and geographic location, with disrespect rates during labor reaching as high as 70% in some studies [23–27]. Furthermore, qualitative studies describe a mix of respectful and disrespectful practices, influenced by facility culture, space and infrastructural constraints, bed-sharing, limited family involvement, restricted birth positions, cultural insensitivity, staff shortages, work overload, neglect, unexpected informal payments, retention for failure to pay the bill, lack of provider oversight and accountability [28–31].

Dar es-salaam, one of the fastest growing cities in Africa is designated as a priority region for healthcare investment in Tanzania’s National strategic plan [32,33]. Despite near-universal institutional births and antenatal care, maternal and perinatal mortality in Dar es Salaam remain nearly double the national average [8,34,35]. A study in an urban health facility in Dar es Salaam in 2013, reported a 15% prevalence of mistreatment [25]. Studies in Tanzania have highlighted important client- and provider-level determinants of RMC, but structural factors like shared beds in low-resourced maternity units remain underexplored [25]. This is despite observations that disrespect often stem from both interpersonal factors and structural constraints [36,37].

This study builds on a decade-long quality improvement initiative by CCBRT and Dar es Salaam regional health authorities, which enhanced Basic and Comprehensive Emergency Obstetric care, but did not assess women’s experiences of mistreatment [38]. To address this gap, we drew on existing tools and collaborated with key stakeholders in a cocreation and validation process, resulting in a contextual 25-item Respectful Maternity Care Tool (RMC-T) designed for integration into quality improvement efforts [12]. The RMC-T was further applied in the PartoMa scale-up study to improve birth outcomes through contextualized clinical practice guidelines and on-the-job training in five health facilities in Dar es Salaam [39]. In this paper, we assess the prevalence and factors associated with women’s experiences of RMC and mistreatment in four urban maternity units in Dar es Salaam.

## Methods

### Study context and setting

This cross-sectional study was conducted from 11 December 2021 to 24 February 2022 in four high-volume government-owned maternity units in Dar es Salaam, one of Africa’s rapidly urbanizing cities with nearly 6 million residents and a 5.4% growth rate [40]. Institutional births in Dar es Salaam are nearly universal (99%), and most pregnant women (98%), make at least one antenatal clinic visit [24]. However, government-subsidized maternity care is constrained by limited resources and women are expected to contribute towards costs for birth kits, laboratory tests and certain medications [38,41].

The study facilities included three regional referral hospitals and one municipal maternity hospital, which collectively recorded 27,450 births in 2020, with individual facilities reporting between 5,803 and 8,384 births [42]. This represented approximately 26.8% of the 102,311 births in the region (Dar es Salaam Regional Annual Report). While relatives may accompany a woman to hospital, they are not allowed to be present during labor and birth [12]. The formative phase revealed women’s concerns about bed-sharing in antenatal and postnatal wards; however, it was not reported during birth [12]. At the study hospitals, medical pain relief during labor is not routinely provided. After birth, paracetamol may be offered if women complain of pain. Women undergoing cesarean section are typically expected to receive opioids intraoperatively, with additional pain management offered postoperatively. Additional details on the study setting are available in previous publications by this author group [12,39,42–44]

### Participant inclusion and consent

Eligible participants were postnatal women aged 15 years or older, who had given birth at a study facility. Informed verbal consent was obtained from all participants. A standardized script outlining the research purpose, procedures, participant rights, anonymity, voluntariness, and the right to withdraw consent at any time was read to the participants prior to the interviews, with verbal consent documented on the data recording form. Verbal consent was selected because it was feasible, culturally acceptable, and permitted by the national ethical guidelines for non-clinical trials [45]. Tanzanian law and research guidelines recognize pregnant girls aged 15–17 as mature minors, enabling them to provide independent informed consent and assent if no harm is anticipated, with their inclusion being crucial due to young maternal age being a known risk factor for the study outcome [14,45,46]. To capture experiences of care provided within the study hospitals, we excluded women who were referred after birth or had been in labor for less than two hours in our facilities, as their responses would primarily reflect care received elsewhere. Women who were severely ill or unconscious during birth were also excluded for ethical and methodological reasons, consistent with standard research practice, to ensure meaningful informed consent.

### Data collection tool

The full description of the co-creation and validation process of the 25-question RMC Tool is detailed elsewhere [12]. A summarized version of the tool is included as Suppl. Table 1.

### Sample size estimation

We used a 5% prevalence of physical abuse as previously reported in Dar es Salaam, to calculate the required sample size [25]. A minimum of 73 participants per health facility was needed for 5% precision and 95% confidence. To account for the additional self-administered data collection option requested by facility leadership, we approximately doubled the sample size. With an expected response rate of 90%, the final study sample was set at 200 participants per health facility.

### Participant recruitment procedures

Data was collected by five Swahili-speaking research assistants (RAs) under supervision by the PI and co-PI. All the RAs were over 25 years old and not involved in maternity care at the study sites. The RAs received three days of training in research ethics, participant recruitment, and conducting sensitive interviews. Pre-discharge exit interviews, lasting 20–30 minutes were conducted in Swahili with all eligible, consenting women until the target sample size was reached in each facility. At the study facilities, women are discharged once daily after morning rounds, ensuring that all women, regardless of the time of birth, could be included. The RAs administered the first part of the tool to all participants which consisted of eligibility screening, informed consent and collection of sociodemographic and obstetric characteristics. This was followed by a phase where RAs administered the 25-item questionnaire to 100 women per facility, after which an additional 100 women per facility self-administered the paper-based questionnaire. Although a private side room was prepared for interview, most women preferred to be interviewed in the ward, and their preferences were accommodated [12]. Women were not provided with any stipend to participate in the interview. After data collection, RAs entered the data into a KoBotoolbox smartphone app. All questionnaires were carefully anonymized, securely managed by the research assistants, and overseen by the PI to ensure confidentiality and data protection.

### Variables and measurement

Independent variables included sociodemographic and maternal characteristics such as maternal age, education level, parity, mode and time of birth. Birth times were categorized according to nurse shifts: morning (8 am–3:59 pm), afternoon (4 pm– 7.59pm) and night (8 pm–7:59 am). Other variables included self-reported birth complications and the baby’s condition after birth. The dependent variables were the 25 items measured by the RMC-T (Suppl. Table 1). These 25 variables were recategorized into nine composite binary (yes/no) indicators grouped into the following themes: 1. effective communication, 2. satisfaction with care, 3. supportive care, 4. dignity and respect (referring to the woman not reporting any of the 11 types of mistreatment (including physical, verbal, and sexual abuse; neglect; discrimination/stigma; lack of privacy; unconsented care; post-birth clean-up; informal payments; and denial of care), 5. ‘one woman, one bed’ (no bed sharing), 6. newborn respect, 7. pain relief, 8. conducive infrastructure (adequate staff, clean environment and toilets, adequate medications and equipment) and 9. birth companionship (Suppl. Table 2). These variables broadly align with the WHO framework for maternal and newborn healthcare quality, addressing communication, dignity and respect, and supportive care [47]. The composite variables with positive wording were emphasized to align with the RMC-T’s purpose of supporting quality improvement. For example, we reported the proportion of women receiving pain relief (instead of the negative phrasing, ‘lack of pain relief’) and reversed ‘bed-sharing’ to ‘one woman, one bed’ to avoid the double negative association with ‘no bed-sharing.’

A ‘Yes’ response was defined using ‘AND’ logic, where all sub-questions of a composite outcome indicated the presence of positive and absence of negative care (RMC). A ‘No’ response was generated if any sub-question in a composite outcome indicated the absence of positive or presence of negative care. A ‘No’ response was generated using ‘OR’ logic. ‘Don’t know’ responses were treated as missing data (Suppl. Table 2).

### Statistical data management and analysis

Data were transferred to Stata Corp, Texas-USA, version 14.0 for cross-checking, data cleaning and analysis. Descriptive statistics, including means and standard deviations, frequencies and percentages were used to describe participant’s socio-demographic and maternal characteristics. The prevalence of each of the measured 25 items was calculated. Bivariate analyses, using either Chi-square or Fisher’s exact test, measured associations between sociodemographic characteristics and the composite RMC indicators. Multivariable logistic regression models were fitted to identify associated factors of RMC for eight of the nine composite outcomes. Birth companion ship was excluded due to local practice limitations and concerns discussed previously [12]. Odds Ratios (ORs) were calculated with 95% confidence intervals and statistical significance was set at *p* < 0.05. OR < 1 points towards an unfavorable outcome or disrespectful care. Results were presented using tables and figures.

### Management of missing data

The data from the researcher-administered and self-administered questionnaires were comparable except for differences in the ‘don’t know’ responses [12]. Overall, ‘don’t know’ responses were low (1.3%), with the self-administered group reporting higher rates (2.5%) than the researcher-administered group (0.1%). These responses were treated as missing data, which remained within the acceptable limit of less than 5% [48]. Pooled data were used for further analysis.

### Ethics

Permission letters to conduct the interviews were obtained from the management of each participating hospital, the local government, and the Ministry of Health, Tanzania. Ethical approval was granted by the National Health Research Ethics Review Committee (NatHREC) of the National Institute for Medical Research in Tanzania (NIMR/HQ/R.8c/Vol. I/2539). A standardized script outlining the research purpose, procedures, participant rights, and voluntariness was read to the participants prior to the interviews, with verbal consent documented.

## Results

Out of 838 postpartum women interviewed, the majority (660, 78.8%) were aged between 20 to 34 years, and 71 (8.5%) were teenagers. Secondary education was completed by 384 (45.8%) participants and 410 (48.9%) had given birth during the 12-hour night shift. Of all participants, 128 (15.3%) were single, while the remaining were married or cohabiting. Among them, 29 (3.5%) experienced an early perinatal death (Table 1).

**Table 1.** Sociodemographic and pregnancy characteristics of 838 postnatal women in four urban hospitals in Dar es Salaam, Tanzania.

Variable	Frequency	Percentage
Maternal age (years)		
15–19	71	8.5
20–34	660	78.8
Above 34	107	12.8
Mean age (SD)	26.6	5.9
Education level		
None/incomplete primary school	53	6.3
Completed Primary school	401	47.9
Completed secondary school	325	38.8
University/college	59	7.0
Marital status		
Single	128	15.3
Married	535	63.8
Cohabiting (none of the above)	175	20.9
Number of births (including index birth)		
Para 1	336	40.1
Para 2–4	460	54.9
Para 5–9	42	5.0
Referral status		
Referred from another facility	151	18.0
Came directly from home	687	81.9
Mode of birth		
Vaginal birth	692	82.6
Cesarean birth (CS)	140	16.7
Vacuum birth	6	0.7
Time of birth		
Morning shift	236	28.2
Evening shift	176	21.0
Night shift	410	48.9
Missing	16	1.9
Birth complications		
Yes	203	24.2
No	635	75.8
Condition of baby		
Baby well	752	89.7
Baby sick	57	6.8
Baby died	29	3.5

” >Due to rounding up, percentages may not add up to 100. Except for time of birth, there were no missing data in any of the other variables. Birth complication refers to women’s self-reported complications such as high blood pressure, prolonged labour/big baby/Cephalo-Pelvic Disproportion, malpresentation (breech, shoulder presentation), Premature rupture of membranes, Postpartum Hemorrhage, perineal tears/episiotomy, abruptio placenta and anaemia.

**Figure 1.**
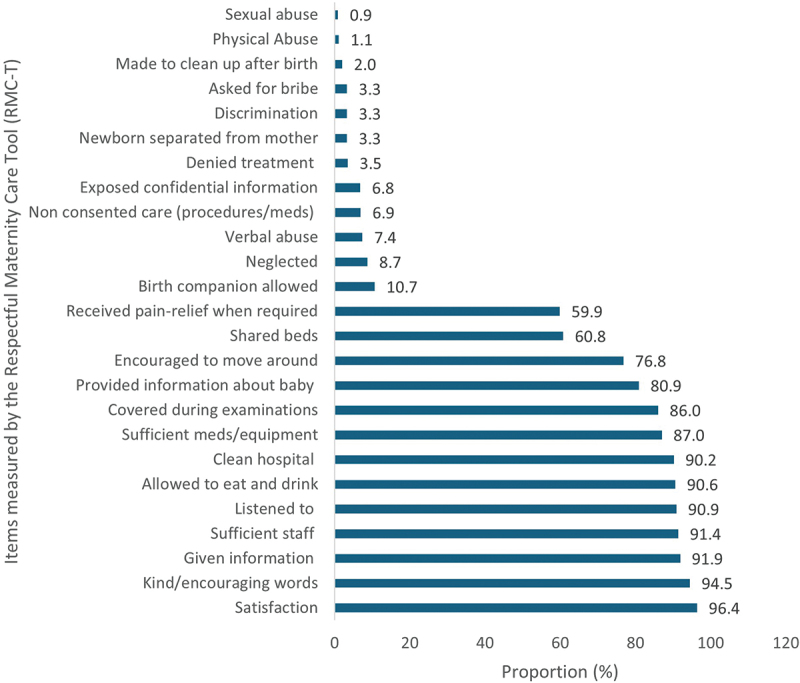
Items measured by the 25-item RMC-Tool among 838 postnatal women at four urban hospitals in Dar es Salaam, Tanzania.

**Figure 2.**
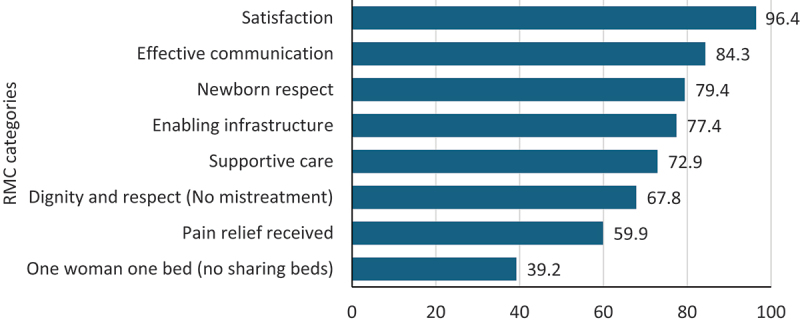
Prevalence of respectful maternity care (RMC) based on composite indicators among 838 postnatal women across four urban hospitals in Dar es Salaam, Tanzania.

Figure 1 illustrates the prevalence of women’s satisfaction with care and experiences of care, as measured by the RMC-T, highlighting the proportions of the 11 types of mistreatments. Figure 2 shows the proportions of composite RMC categories.

Composite RMC indicators were derived from recategorizing the 25-items in the RMC-T into the following categories: *Effective communication* (Kindness, listened to, given information). *Supportive care* (Encouraged to move around, allowed to eat and drink). *Dignity and respect /No Mistreatment* (Consented care, no neglect, no discrimination/stigma, no verbal abuse, no physical abuse, no sexual abuse, confidential care related to information, privacy (body covered, curtains), not made to clean up after-birth, no informal payments, no denial of treatment due to inability to pay). *Enabling infrastructure* (Adequate staff, clean environment and toilets, adequate medications and equipment). *Newborn respect* (mother given information about newborn, no mum-baby separation). *One woman one bed* (reversal of bed sharing), *Satisfaction* and *pain relief received* were single questions from the RMC-T.

Satisfaction was reported by 96.4% (793/823) women. Additionally, 84.3% (689/817) reported effective communication. However, 60.8% (503/827) shared hospital beds, 59.9% (488/815) received pain relief following procedures such as repair of perineal tears and cesarean section, and 10.7% (89/829) reported that they had a birth companion. Of all women, 32.2% (253/785) experienced one of the 11 types of mistreatments, with no covering during examination (14.0%), neglect (8.8%), verbal abuse (7.5%) and procedure without consent (6.9%) being the most frequent types. Bribes were reported by 3.3% of participants, while 3.5% reported being denied treatment due to inability to pay (Figure 1).

**Figure 3.**
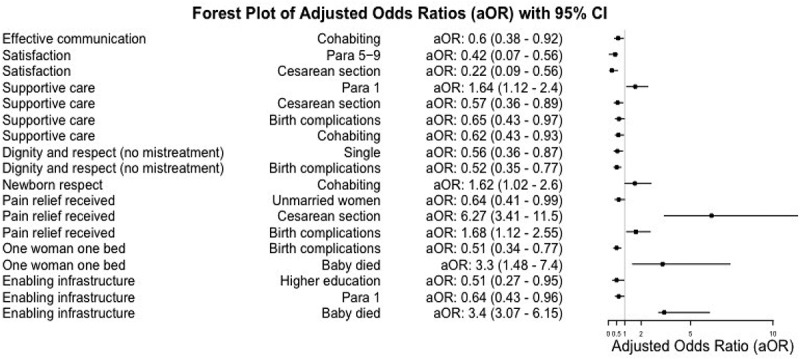
Significant factors associated with respectful maternity care (composite indicators) among 838 postnatal women in four urban hospitals in Dar es Salaam, Tanzania.

Women with grand multiparity (aOR 0.42; 95% CI 0.07–0.56) and those who had caesarean births (aOR 0.22; 95% CI 0.09–0.56) were less likely to report satisfaction with their care. Meanwhile, dignity and respect was significantly less frequent among single women (aOR 0.56; 95% CI: 0.36–0.87) and those with childbirth complications (aOR 0.52; 95% CI: 0.35–0.78). The likelihood of self-reported complications during childbirth was significantly reduced when each woman had their own bed (aOR 0.51; 95% CI: 0.34–0.77) (Figure 3).

Composite RMC indicators resulted from recategorizing the 25-items in the RMC-T. Items were reversed towards Respectful care. OR > 1 suggestive of RMC and OR  < 1 suggestive of lack of RMC.

First-time pregnant women were more likely to report supportive care (aOR 1.64; 95% CI 1.12–2.41), but less likely to view the healthcare infrastructure as adequate (aOR 0.64; 95% CI 0.43–0.96). Additionally, women with higher education levels were also less likely to consider the infrastructure adequate (aOR 0.51; 95% CI 0.27–0.95). Women with cesarean birth were less likely to receive supportive care (aOR 0.57; 95% CI 0.36–0.89).

## Discussion

This study illustrates the complexity of providing RMC in under-resourced urban settings highlighting the unpredictable interplay between care practices, structural constraints, patient experiences and adverse outcomes, a dynamic not unique to this study setting and described as a ‘wicked problem’ [37,49].

The coexistence of high satisfaction ratings (96.4%) with reported mistreatment (32.2%) and two-thirds of women sharing hospital beds is striking. This mixed picture is also documented in other LMIC studies [29]. In settings where deference, politeness, or gratitude are customary, women may report satisfaction despite negative experiences [50].

Studies report that satisfaction and care experience are related but distinct concepts [51,52]. The woman-provider relationship is a key factor influencing satisfaction, and women’s positive perception of effective provider communication (84.3%) may account for the high satisfaction scores reported in this study [52]. Other factors influencing satisfaction scores includes cultural and societal influences which do not always align with Western frameworks, as well as socio-economic status, women’s vulnerability, care expectations, awareness of childbirth rights, and normalization of sub-standard practices [52]. In HICs, where women are far more aware of their rights, there is a stronger correlation between satisfaction and care experiences [50,53–55]. Studies recommend assessing both care satisfaction and specific experiences, with qualitative methods providing deeper insights [55]. However, client interests are best served by balancing the benefits of costly time-consuming approaches and ensuring feedback effectively informs decision-makers [51].

Women in our study rated effective communication and confidentiality higher than reported in India, Ghana, Ethiopia and other regions of Tanzania [56–58]. The effective communication score is plausibly a reflection of ongoing national efforts to strengthen RMC, the development and dissemination of national guidelines on respectful and compassionate care, and a decade-long quality improvement initiative at the study facilities [38,59]. Similar high positive findings were reported in the 2022 Demographic and Health Survey [24].

The prevalence of physical abuse in this study was 1.1%, lower than previous reports from Dar es Salaam (5% in 2013) and the 2022 demographic survey (13%), but similar to rates observed in Kenya, Ghana, and India (3%) [24,25,56]. Although reports of bribes and treatment denial were low in our study, they contrast with Binyaruka’s finding that 27% of healthcare workers engaged in informal payments, acknowledging that bribe levels may vary across facilities and regions [60].

Understanding the context and variable composition is crucial for meaningful cross-study comparisons, as variation in mistreatment rates often arise from differences in measurement tools, variable description, settings, sampling and data collection timing [61]. Higher rates are typically reported in direct observations, home-based interviews, and when more mistreatment items or infrastructural constraints are included [26,27]. For instance, a rural Tanzanian survey with 13 items, including infrastructural items, reported 73.1%, mistreatment, while studies excluding these factors found lower rates: 19.5% (rural) and 15% (urban) [25–27].

Severe overcrowding, reflected in bed-sharing in early labor and in the postnatal wards, affected two-thirds of women in our study. While more common in urban areas, it also occurs in rural Tanzania [24,27]. Although bed-sharing was not directly linked to mistreatment, it was associated with complications, which were in turn linked to mistreatment, a pattern widely reported in LMICs [53,62,63]. Overcrowded wards and staff shortages can exacerbate skill gaps, delay recognition and management of complications, contribute to workforce burnout, stress, and reduced capacity to provide timely, standard care, and contribute to mistreatment [64–67].

### Strengths and limitations

The study’s strengths include a large sample size across four major referral hospitals and the use of a validated contextualized tool for rapid, low-cost, real-time measurement of women’s birth experiences, highlighting gaps and providing opportunities for targeted quality improvement. The study has, however, several limitations, including underreporting due to timing and venue of exit interviews, which can introduce information and social desirability bias [52,61]. Facility-based recruitment may introduce selection bias, though as noted in previous work, fewer than seven women refused participation, mainly due to a crying baby, indicating minimal impact on findings [12]. The purposeful selection of health facilities limits the generalizability of the findings to large urban referral hospitals, recognizing that care patterns in rural facilities, smaller hospitals and private sector care may differ substantially. Additionally, the cross-sectional design without a comparison group restricts causal inferences. We did not model birth companionship, a key determinant of respectful care, due to existing facility policies that restrict it and concerns that some women may have misunderstood birth companionship, confusing it with being escorted to the hospital, a misconception also found in other studies [27]. Unmeasured confounders, such as socio-economic status, provider characteristics, cultural practices or prior healthcare experiences, may have influenced the results, though the magnitude and direction of their effect remain uncertain [63]. Furthermore, the study relied solely on women’s perceptions to assess birth complications, staffing, and supplies, limiting definitive conclusions about associations with infrastructure or health outcomes [68].

Finally, the RMC-T prioritizes practicality over measurement precision [12]. Readers are referred to the cited reference for a full discussion of strengths and limitations of the tool [12]. The nine composite outcomes aligned with the WHO typology framework, offer valuable insights but may obscure nuances and challenge comparison across studies

### Clinical implications of research findings

High satisfaction scores coexisting with high mistreatment highlight the limitation of using exclusively satisfaction surveys to collect customer feedback and the importance of including assessment for specific care experiences. Routine administration of the RMC-T to randomly selected postnatal women can generate sufficient and timely data to guide facility improvements, enhance awareness of childbirth rights, and reinforce accountability among clients, providers, and health leaders [12]. Integrating rights-based messaging into antenatal health talks could offer a quick win, with follow up measurement to assess changes after education.

Providing both interviewer-administered and self-administered measurement options ensures inclusivity for varying literacy and abilities while balancing the need for privacy and allows women to choose the method most comfortable and accessible to them. Further implementation research is needed to assess the feasibility and effectiveness of integrating routine RMC measurements into facility-level quality improvement.

Bed sharing, rarely included in common RMC tools and inconceivable in high-income settings, was the predominant complaint during the tool’s formative phase, with women expressing distress, humiliation, and infection concerns [12,16,25,69–71]. In the context of global urbanization and efforts to increase institutional births, challenges such as bed-sharing and staff shortages may intensify, undermining the provision of safe and respectful care [35,72]. It is crucial not to turn laboring women away but to prioritize health system redesigns that alleviate congestion, redistribute workloads, and enhance capacity [44,73,74]. Decongestion would also create an enabling environment for birth companionship, an evidence-based approach to promoting physiological birth and improving care experiences [75]. Measuring bed-sharing can generate evidence to advocate for one woman per bed, protecting safety and dignity in childbirth.

The challenge of managing obstetric complications in overcrowded wards, and its effect on care experiences, should not be underestimated. Alongside addressing workforce shortages, training programs should also emphasize triaging, teamwork, stress management, and rights-based care [39,76].

Although Tanzania has advanced in promoting RMC, any form of mistreatment must not be accepted. Lessons from national programs, such as HIV, show that even complex challenges can be transformed into effective national programs. With sustained investment, training, supervision, and community engagement, RMC can similarly be strengthened and institutionalized [44,77,78].

## Conclusion

High satisfaction scores, alongside mistreatment, bed-sharing, and lack of birth companionship, highlight the need to raise awareness of rights-based care among women, providers, and leaders. As urban expansion and increased demand for care outpace healthcare investments, it is crucial to address structural constraints, bed-sharing and staff shortages. Strengthening provider training on respectful care and implementing routine RMC measurements can promote accountability, enhance clinical outcomes, and improve women’s experiences of care.

## Supplementary Material

STROBE checklist_One woman one bed.doc

Supplementary_Files_de.docx

## Data Availability

The data supporting the findings of this study, including deidentified participant data, are available from the corresponding author, [BSD], upon reasonable request. The current ethical approval does not permit public dissemination of the data.
