# Supplementary material for: ‘One woman, one bed’: prevalence and factors associated with women’s experiences of respectful birth in urban Dar es Salaam, Tanzania – across-sectional survey
Source: Glob Health Action. 2025 Oct 24;18(1):2568295. doi: 10.1080/16549716.2025.2568295 (PMC12557818; doi:10.1080/16549716.2025.2568295)
Supplement: STROBE checklist_One woman one bed.doc [file ZGHA_A_2568295_SM3626.doc]

STROBE Statement/checklist for observational studies- ‘One woman, one bed’: prevalence and factors associated with women’s experiences of respectful birth in urban Dar es Salaam, Tanzania – a cross-sectional survey’.

| Item | Description/Details | Compliance statement (Yes/No /Partial) |
| --- | --- | --- |
| 1. Title and abstract | (*a*) Indicate the study’s design with a commonly used term in the title or the abstract | Yes |
|  | (*b*) Provide in the abstract an informative and balanced summary of what was done and what was found | Yes |
| 2. Introduction Background/rationale | Explain the scientific background and rationale for the investigation being reported | Yes |
| 3. Introduction Objectives | State specific objectives, including any prespecified hypotheses | Yes |
| 4. Methods  Study design | Present key elements of study design early in the paper | Yes |
| 5. Setting | Describe the setting, locations, and relevant dates, including periods of recruitment, exposure, follow-up, and data collection | Yes |
| 6. Participants | *Cross-sectional study*—Give the eligibility criteria, and the sources and methods of selection of participants | Yes |
| 7. Variables | Clearly define all outcomes, exposures, predictors, potential confounders, and effect modifiers. Give diagnostic criteria, if applicable | Yes-Page number 7 and Supplementary File 2 |
| 8. Data sources/ measurement | For each variable of interest, give sources of data and details of methods of assessment (measurement). | Yes |
| 9. Bias | Describe any efforts to address potential sources of bias | Yes |
| 10. Study size | Explain how the study size was arrived at | Yes |
| 11.Quantitative variables | Explain how quantitative variables were handled in the analyses. If applicable, describe which groupings were chosen and why | Yes |
| 12.Statistical methods | (*a*) Describe all statistical methods, including those used to control for confounding | Yes |
|  | (*b*) Describe any methods used to examine subgroups and interactions | Yes |
|  | (*c*) Explain how missing data were addressed | Yes |
|  | *Cross-sectional study*—If applicable, describe analytical methods taking account of sampling strategy | Yes |
|  | (*e*) Describe any sensitivity analyses | Yes- partial |
| 13. Results  Participants | (a) Report numbers of individuals at each stage of study—eg numbers potentially eligible, examined for eligibility, confirmed eligible, included in the study, completing follow-up, and analysed | Yes |
|  | (b) Give reasons for non-participation at each stage | Yes-partial |
|  | (c) Consider use of a flow diagram | Not applicable |
| 14.Descriptive data | (a) Give characteristics of study participants (eg demographic, clinical, social) and information on exposures and potential confounders | Yes |
|  | (b) Indicate number of participants with missing data for each variable of interest | Yes |
| 15.Outcome data | *Cross-sectional study—*Report numbers of outcome events or summary measures | Yes |
|  |  |  |
| 16. Main Results | (*a*) Give unadjusted estimates and, if applicable, confounder-adjusted estimates and their precision (eg, 95% confidence interval). Make clear which confounders were adjusted for and why they were included | Yes_ In supplementary File (Tables 3-6) |
|  | (*b*) Report category boundaries when continuous variables were categorized | Not applicable |
|  | (*c*) If relevant, consider translating estimates of relative risk into absolute risk for a meaningful time period | No |
| 17. Other results | Report other analyses done—eg analyses of subgroups and interactions, and sensitivity analyses | Yes- Main/Suppl. tables |
| Discussion |  |  |
| 18. Key results | Summarise key results with reference to study objectives | Yes |
| 19. Limitations | Discuss limitations of the study, taking into account sources of potential bias or imprecision. Discuss both direction and magnitude of any potential bias | Yes |
| 20. Interpretation | Give a cautious overall interpretation of results considering objectives, limitations, multiplicity of analyses, results from similar studies, and other relevant evidence | Yes |
| 21. Generalisability | Discuss the generalisability (external validity) of the study results | Yes |
| 22. | Give the source of funding and the role of the funders for the present study and, if applicable, for the original study on which the present article is based | Yes |
