# Supplementary material for: ‘One woman, one bed’: prevalence and factors associated with women’s experiences of respectful birth in urban Dar es Salaam, Tanzania – across-sectional survey
Source: Glob Health Action. 2025 Oct 24;18(1):2568295. doi: 10.1080/16549716.2025.2568295 (PMC12557818; doi:10.1080/16549716.2025.2568295)
Supplement: Supplementary_Files_de.docx [file ZGHA_A_2568295_SM3625.docx]

| **Supplementary Table 1: Variables measured in the Respectful Maternity Care Measurement Tool** | |
| --- | --- |
|  | Treated kindly |
|  | Given a chance to ask questions and listened to your concerns and wishes |
|  | Explain examinations or procedures/or giving you medication |
|  | Receive any medication or procedures without permission/consent |
|  | Neglected or ignored you |
|  | Stigma and discrimination |
|  | Birth companionship |
|  | Encouraged to walk/move around during labor |
|  | Allowed to eat and drink when you wanted to or when you were thirsty/hungry |
|  | Was pain medication given during and after painful procedures |
|  | Verbal abuse |
|  | Physical abuse |
|  | Experienced flirting comments, inappropriate sexual language or request for sex |
|  | Shared your information out loud such that others could hear |
|  | During examinations were you always covered up with a cloth or curtain |
|  | Did you have to share a bed /or mattress with another woman or women |
|  | Made to clean up your own blood, birth ﬂuid from the birthing area/bed |
|  | Asked for a bribe, informal payment, or gift |
|  | Receive information about the condition of your baby |
|  | Baby separated from you after birth without information/explanation |
|  | Denied treatment due to inability to pay |
|  | Enough health staff in the facility to care for you |
|  | Clean facility, toilets and washing facilities |
|  | Enough medicines and supplies in the facility to care for you |
|  | Would you recommend this facility to other women for giving birth |
| Note: This This is a summarized version of the 25 RMC-specific questions. The original tool is available *from: Sequeira Dmello et al. Global Health Action, 2024.doi.org/10.1080*/16549716.2024.2403972 [*http://creativecommons.org/licenses/by/4.0/*](http://creativecommons.org/licenses/by/4.0/) | |

| **Supplementary Table 2: Recategorizing the 25- RMC-T questions into nine new composite RMC variables** | |
| --- | --- |
| **New variable (name)- yes/no** | Measurement description |
| 1. Effective communication   (measured using 3 items) | Response was categorised as ‘yes’ IF women responded YES to the following three questions:   1. ‘Spoke kind/encouraging words during ward stay’ AND 2. ‘Listened to you /allowed you to ask questions’ AND 3. ‘Gave you information about procedures’.   Only those who scored ‘*Y*es’ to all these three items were classified as YES (having effective communication). Those who answered ‘No’ to any of these three items were categorized as ‘No’ (effective communication). |
| 1. Satisfaction *(with care)*   (measured using 1 item) | Relabelled the variable from the original question which read ‘would you recommend this facility to your loved ones or family?  The original yes/no responses apply. |
| 1. Supportive care   (measured using 2 items) | Response was categorised as ‘*Y*es’ if women responded yes to   1. Encouraged to walk/move around during labor AND 2. Allowed to eat and drink   Only those who scored ‘*Y*es’ to these two items were classified as having received supportive care and those who answered ‘No’ to any of these two items were classified to no supportive care. |
| 1. Dignity and respect (No mistreatment) *   (measured using 11 items) | The option ‘*Y*es’ reflects treated with ‘dignity and respect’, implying no incident of mistreatment was reported.  Women were categorized as ‘*Y*es’ to variable dignity and respect’ ONLY if they experienced none of the incidents of mistreatment [they answered no to items (a-j), and also answered yes to item k]   1. Non consented care (question 4); AND 2. Felt ignored/neglected (question 5); AND 3. Stigma or Discrimination (question 6); AND 4. Verbal abuse/shouted at/threatened (question 11); AND 5. Physical abuse (question 12); AND 6. Sexual abuse (question 13); AND 7. Non-confidential care (personal information) (question 14); AND 8. Instructed to clean up immediately after birth (question 17); AND 9. Asked for a bribe, informal payment or gift (question 18); AND 10. Denied treatment due to inability to pay (question 21) AND 11. Being covered, curtains (bodily privacy) (question 15) |
| 1. No sharing beds (one woman one bed)   (measured using 1 item) | This response was extracted from question 16 in the RMC-T (original question was ‘at any time did you share beds or a mattress with another woman or women?).  Those who answered ‘No’ had given the favorable response, meaning they did not share beds, but were categorized as ‘yes’ in the recoding of variables positively. |
| 1. Newborn respect   (measured using 2 items) | If women answered, ‘*Y*es’ to ‘Received information about condition of baby (question 19); AND answered ‘No’ to the question ‘separated from mother without information’ (question 20), they were categorized as a ‘Yes’.  while those who answered ‘No’ to question 19 and/or yes to question 20, or no to both questions or Yes to both questions were categorized as ‘No’ |
| 1. Pain relief received   (measured using 1 item) | This question was unchanged from the original question 10. The original yes/no responses apply. |
| 1. Enabling infrastructure   (measured using 3 items) | Women who answered yes to:   1. ‘Do you think there were enough staff in the facility to care for you?’ (question 22); AND 2. ‘Were hospital environment and toilets clean’, (question 23) AND 3. ‘Do you think there were enough medicines and supplies in the facility to care for you?’.   Only if all three responses were yes, they were categorized as ‘*Y*es’. And those who answered ‘No’ to any of these three questions, or all three questions were categorized as ‘No’ |
| 1. Birth companionship** (measured using 1 item) | No change to this question. The original yes/no responses apply. |
| * If women experienced even one of the (a-k) incidents of mistreatments, they were categorized as ‘No dignity and respect’ (implying that they had experienced at least one incident of mistreatment). Dignity and respect are WHO language. We reversed the negative term (mistreatment) into a positive term in the creation of this new variable, so that all indicators would be phrased in a positive language signifying RMC and to avoid the double negative if we used the phrase No mistreatment.  **Birth companionship was considered a standalone indicator (and not a sub-item of supportive care). At the time of this study, it was not routine practice in the study hospitals. This study provides baseline information on birth companionship.  Don’t know responses were considered missing data and excluded. | |
